# Challenging the paradigm of metabolic exclusivity: coexistence of methanogenesis and sulfate reduction in oil reservoirs

**DOI:** 10.1128/aem.02172-25

**Published:** 2026-01-27

**Authors:** Shanquan Wang, Yi Su

**Affiliations:** 1 School of Environmental Science and Engineering, Environmental Microbiomics Research Center, Guangdong Provincial Key Laboratory of Environmental Pollution Control and Remediation Technology, Southern Marine Science and Engineering Guangdong Laboratory (Zhuhai), Sun Yat-Sen University 26469 , Guangzhou, China; Michigan State University, East Lansing, Michigan, USA

**Keywords:** methanogenesis, sulfate reduction, microbial competition, oil reservoir, metabolic coexistence

## Abstract

The prevailing dogma in microbial ecology holds that sulfate-reducing microorganisms (SRMs) outcompete methanogenic archaea for common substrates (e.g., H_2_/formate and acetate), leading to the mutual exclusion of sulfate reduction and methanogenesis in sulfate-rich anaerobic environments. This principle underpins models of organic carbon flow to sulfate-respiration-derived CO_2_ in ecosystems like oil reservoirs, where seawater injection introduces high concentrations of sulfate. In an *Applied and Environmental Microbiology* article by S. Beilig, L. Voskuhl, I. Geydirici, L. K. Tintrop, T. C. Schmidt, and R. U. Meckenstock (91:e00141-25, 2025, https://doi.org/10.1128/aem.00141-25), the authors challenge this view by demonstrating coexistence of methanogenesis and sulfate reduction in a sulfate-adapted enrichment culture from an oil reservoir. The authors employ incubation experiments and microbial activity assessment via the reverse stable isotope labeling (RSIL) method to argue for metabolic coexistence, even under conditions thought to favor complete competitive exclusion. This commentary discusses the mechanistic reasons underlying the coexistence and explores the broader implications for predicting microbial activities and interactions. The study compellingly argues that thermodynamic and kinetic arguments alone are insufficient to predict microbial community function, necessitating a more nuanced understanding of microbial interactions in complex environments.

## COMMENTARY

Methanogenic archaea and sulfate-reducing microorganisms (SRMs) are ubiquitous in anoxic habitats and share similar growth conditions, including the carbon sources and electron donors (1, 2). The traditional methanogenic fermentation involving consequent acidogenic fermentation, syntrophic acetogenesis, and methanogenesis is mediated by fermentative bacteria, syntrophic acetogens, and methanogenic archaea, respectively. In this process, the methanogens facilitate the fermentation process by consuming the acetate, H_2_/CO_2_, and formate to improve the thermodynamic conditions. When introducing SRMs into the methanogenic fermentation process, they play similar roles to the methanogens in removing acetate, H_2_/CO_2_, and formate to support their sulfate respiration and cell growth. Therefore, SRMs and methanogens are generally believed to be competitive, and their mediated dissimilatory sulfate reduction and methanogenesis are typically thought to be mutually exclusive.

Compared to the methanogenic archaea, the metabolic flexibility and inhibitive metabolites (sulfide), together with favorable thermodynamics and kinetics, of SRMs enable them to outcompete methanogenesis in the competition for the acetate/H_2_/formate as carbon sources and/or electron donors (3–6). Nonetheless, Beilig et al. (7) reported the coexistence of methanogenesis and sulfate reduction in a sulfate-reducing enrichment culture derived from an oil reservoir. The authors used incubation experiments and microbial activity assessment via the reverse stable isotope labeling (RSIL) method to confirm the coexistence of the two competitive processes under conditions with high sulfate concentrations. These results challenged the traditional belief that exclusive methanogenesis and sulfate reduction processes exist.

**Fig 1:**
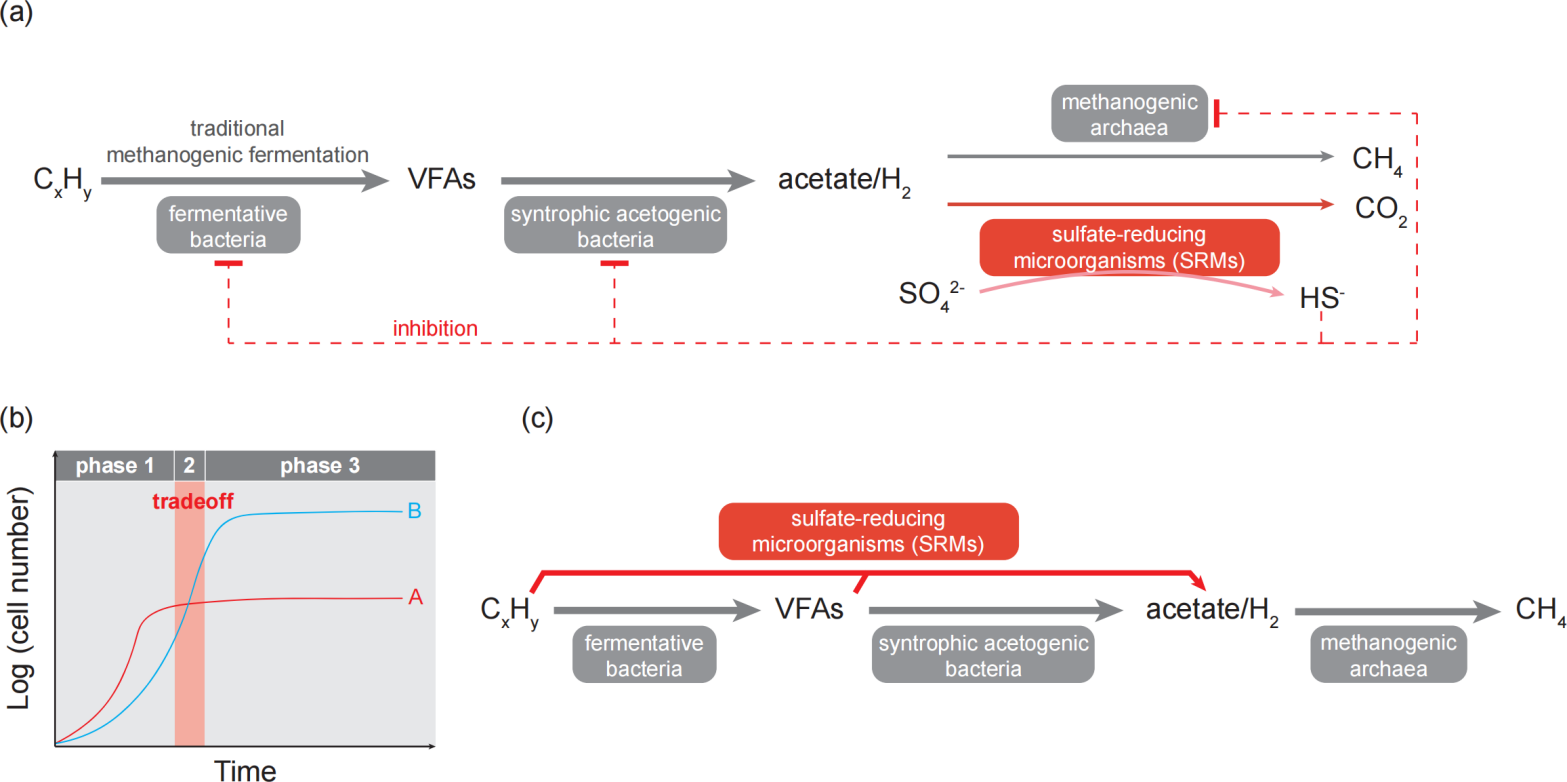
Proposed mechanisms for the coexistence of sulfate-reducing microorganisms (SRMs) and methanogens. (**a**) Introduction of SRMs into the methanogenic fermentation process; (**b**) the rate/yield tradeoff-based growth curves of fast-growing population A with low cell density (red line) and slow-growing population B with high cell density (blue line); and (**c**) metabolic flexibility of SRMs enables complementary metabolisms of SRMs and methanogenic fermentation populations.

Beilig et al. proposed several compelling, yet speculative, mechanisms to explain the observed coexistence of methanogenesis and sulfate reduction: (i) spatial and temporal variations (substrate gradient in microbial flocs/biofilm or in plug-flow reactors) allow both methanogens and SRMs to access substrates; (ii) direct substrate-to-methane conversion without requiring syntrophic partners (e.g., alkane-to-methane converting methanogens) (8); (iii) substrate limitations like low concentrations of sulfate and electron donors, which may result in limited-nutrient growth of microorganisms and conversion of acetate to H_2_/CO_2_ by methanogenic *Methanosarcina* (9, 10); (iv) direct interspecies electron transfer between fermenters and methanogens, which allows the methanogens to circumvent the competition between SRMs and methanogens for the acetate, H_2_, and formate; and (v) tolerance of methanogenic fermentation populations (i.e., fermentative bacteria, syntrophic acetogens, and methanogens) to SRM-derived sulfide (Fig. 1a). Nonetheless, the abovementioned potential mechanisms await experimental evidence, and further meta-omics data can help to clarify the involved mechanisms in this study.

Methanogenesis can be frequently observed to coexist with sulfate reduction in freshwater sediments, landfills, and offshore oil reservoirs (2, 5, 6, 11). Similar to the coexistence of SRMs and methanogens, methanogenesis could also coexist with other anaerobic respiration processes. For example, though organohalide-respiring bacteria (OHRB) use acetate and H_2_ as the carbon source and electron donor, OHRB were commonly observed to coexist with methanogens (2, 11, 12). This similar coexistence of methanogenesis with other respiration processes depends on the complicated and dynamic interactions between these functional microorganisms. Therefore, observations and clarification of underlying mechanisms are critical to understand the interactions of methanogenesis and their interacting populations, as well as their mediated element cycles.

In addition to Beilig et al.’s proposed insights into the coexistence of methanogens and SRMs, there could be two other mechanistic models for the coexistence: (i) growth kinetics of the competitive populations; and (ii) metabolic flexibility of the SRMs. The coexistence of competitive populations in specific growth niches depends on the balance of their cell growth kinetics (Fig. 1b). Competitive populations with similar requirements of substrates/nutrients commonly have different growth rates and cell yield, of which cell growth kinetics are in line with the rate/yield trade-off theory (13, 14). As shown in Fig. 1b, competitive populations A and B may exclusively outcompete each other in growth phases 1 and 3, respectively. In contrast, growth phase 2 enables the co-existence of both populations based on the balanced rate/yield tradeoff. Therefore, it is critical to elucidate the cell growth kinetics of the target methanogens and SRMs to achieve their coexistence or exclusive enrichment of methanogens or SRMs.

The metabolic flexibility of the phylogenetically diverse SRMs enables extremely wide distribution of SRMs in varied environments, including the methanogenic oil reservoirs (7, 15–17). In addition to the methanogenesis-competitive dissimilatory sulfate reduction, taxonomically diverse SRMs were reported to be capable of acetogenic fermentation, which produces acetate and H_2_ to support, rather than exclusively compete with, methanogenic archaea (Fig. 1c). For example, *Desulfovibrio vulgaris* Hildenborough, as a model SRM, converts lactate into acetate/H_2_ and sulfate partially into zero valent sulfur (ZVS), which further supports the cell growth of methanogens by providing carbon source/electron donors and by alleviating sulfide-derived inhibition (17, 18). Without direct mechanistic evidence, these proposals remain in the realm of possibility. Despite the limitations of no direct mechanistic evidence, the study by Beilig et al. holds significant ecological importance. It compellingly argues that thermodynamic and kinetic arguments, while foundational, are not absolute predictors of microbial community function in complex and sulfate-rich environments. The work adds to a growing consensus that coexistence is possible and may be facilitated by substrate heterogeneity, metabolic versatility, and the use of non-competitive substrates. In natural complex environments, SRMs and methanogens may integrate the abovementioned and/or yet-to-be-characterized mechanisms to balance their competitive interactions, which warrant future extensive field explorations.

### Conclusion

The work by Beilig et al. serves as a valuable and provocative challenge to the classical paradigm of thermodynamic exclusion in environmental microbiology. It successfully demonstrates that methanogenesis can persist in sulfate-adapted microbial communities. However, the study also highlights the intricate challenges of interpreting inhibitor-based incubation experiments in complex communities and the limitations of 16S rRNA gene amplicon-sequencing data for inferring function. The proposed mechanisms for coexistence are reasonable but require rigorous and direct validation. Ultimately, this commentary underscores that while the study effectively opens the door to a more nuanced understanding of microbial coexistence, walking through it will require the application of sophisticated analytical tools and field studies to illuminate the precise biochemical pathways and interactions at play.
